# Clinical and pathological characteristics of gastric large cell neuroendocrine carcinoma: A report of 2 cases series and literature review

**DOI:** 10.1097/MD.0000000000039851

**Published:** 2024-10-04

**Authors:** Bo Wang, Yuan Si, Yan Dou, Yongcai Li, Zhongxin Liu, Chaokang Huang, Xin Xu

**Affiliations:** a Department of Pathology, Xingtai People’s Hospital, Hebei Medical University Affiliated Hospital, Xingtai, Hebei, P.R. China; b Endoscopic Center, Xingtai People’s Hospital, Hebei Medical University Affiliated Hospital, Xingtai, Hebei, P.R. China; c Department of Computed Tomography and Magnetic Resonance Imaging, Xingtai People’s Hospital, Hebei Medical University Affiliated Hospital, Xingtai, Hebei, P.R. China

**Keywords:** clinicopathologic features, immunophenotype, insulinoma-associated protein 1 (INSM1), large cell neuroendocrine carcinoma (LCNEC), prognosis, stomach

## Abstract

**Rationale:**

Gastric large cell neuroendocrine carcinoma (LCNEC) is a rare, aggressive neuroendocrine carcinoma that arises from the stomach and with high malignancy. Patients with gastric LCNEC usually have a poor prognosis. The standard treatment plan has not been established and its curative effect is poor. The present study described 2 cases diagnosed with gastric LCNEC and reviewed in depth the literature to improve our understanding more about this uncommon tumor and further to provide more experience to diagnose and treat this disease.

**Patient concerns:**

The present study reported 2 cases of gastric LCNEC in male patients aged 51 and 73 years old, respectively. Both patients had epigastric discomfort, pain, acid reflux and heartburn. The medical history was unremarkable.

**Diagnoses:**

The ulcerative lesion located at gastric was examined in the 2 patients by taking the esophagogastroduodenoscopy (EGO), computed tomography (CT) and digital gastrointestinal radiography (GI), that both were suspected gastric malignancy. Endoscopic biopsies of the tumor led to the initial diagnosis of gastric cancer. Postoperative pathological and immunohistochemical examinations of the surgical specimens confirmed that 1 case had mixed adeno-neuroendocrine carcinoma (MANEC) and the other had LCNEC.

**Interventions:**

Both patients underwent surgical resection and received etoposide-cisplatin combination chemotherapy following surgery.

**Outcomes:**

The operation was successful. Both patients had uneventful recoveries following surgery, and had been followed-up regularly. The general condition was satisfactory, and no tumor metastasis was observed at present.

## 1. Introduction

Neuroendocrine neoplasm (NEN) is developing from the enterochromaffin cells in the neuroendocrine tissue throughout the body.^[1]^ Digestive system NEN has been classified by the World Health Organization in 2019 into 2 main categories: neuroendocrine tumor (NET) and neuroendocrine carcinoma (NEC). NET includes the well-differentiated neuroendocrine tumors (G1 and G2), and the poorly differentiated neuroendocrine tumors (G3). NEC includes the small cell neuroendocrine carcinoma (SCNEC) and LCNEC. NEN is a rare tumor, and gastric LCNEC is a much rarer high-grade neuroendocrine carcinoma that tends to have a poor prognosis. During the last years, only a few sporadic cases of gastric LCNEC have been reported. Usually, these tumors are only found at the time of metastasis making them even more difficult to treat. Therefore, we reported 2 patients with gastric LCNEC. The clinical manifestation, pathology morphology, immunohistochemical characteristics, cell proliferative index and prognosis of these patients were summarized in this study, along with an in-depth literature discussion, in order to improve the pathologist accurate diagnosis, the understanding more about this uncommon tumor and further to provide more experience to treat this disease.

## 2. Case report

### 2.1. Case 1

**Figure 1.**
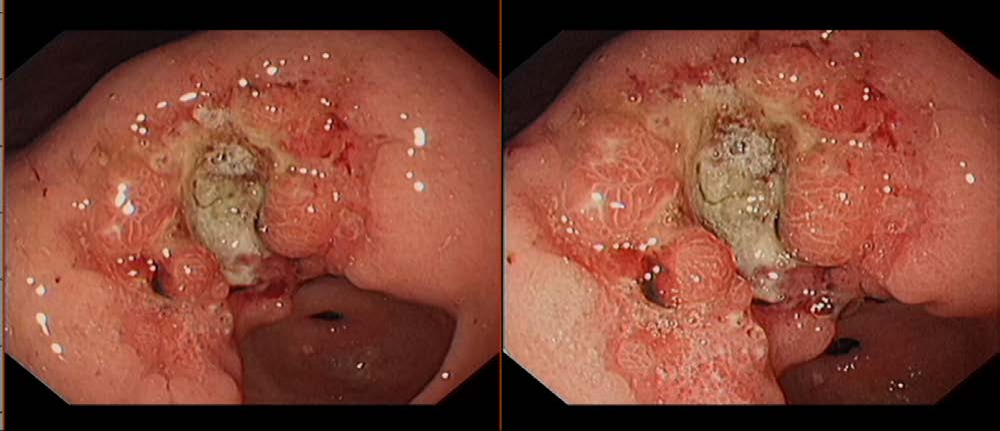
Esophagogastroduodenoscopy (EGD), which was performed, demonstrated an ulcerated mass of the gastric antral.

**Figure 2.**
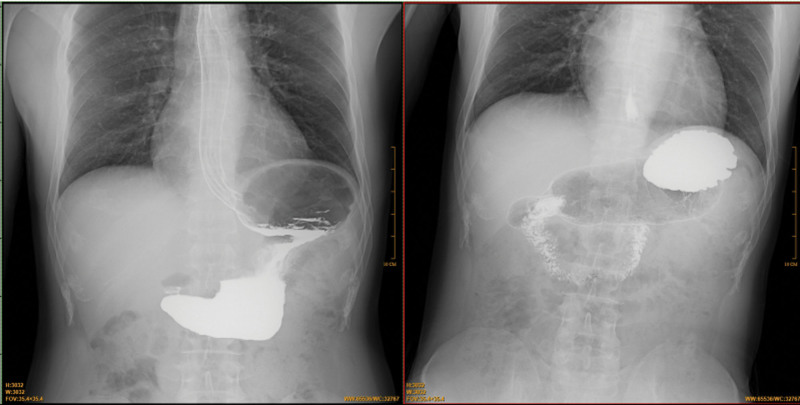
The digital gastrointestinal radiography (GI) showed the gastric sharp angle sign and the mucosal folds were irregular occurred at the lesser curvature of stomach.

**Figure 3.**
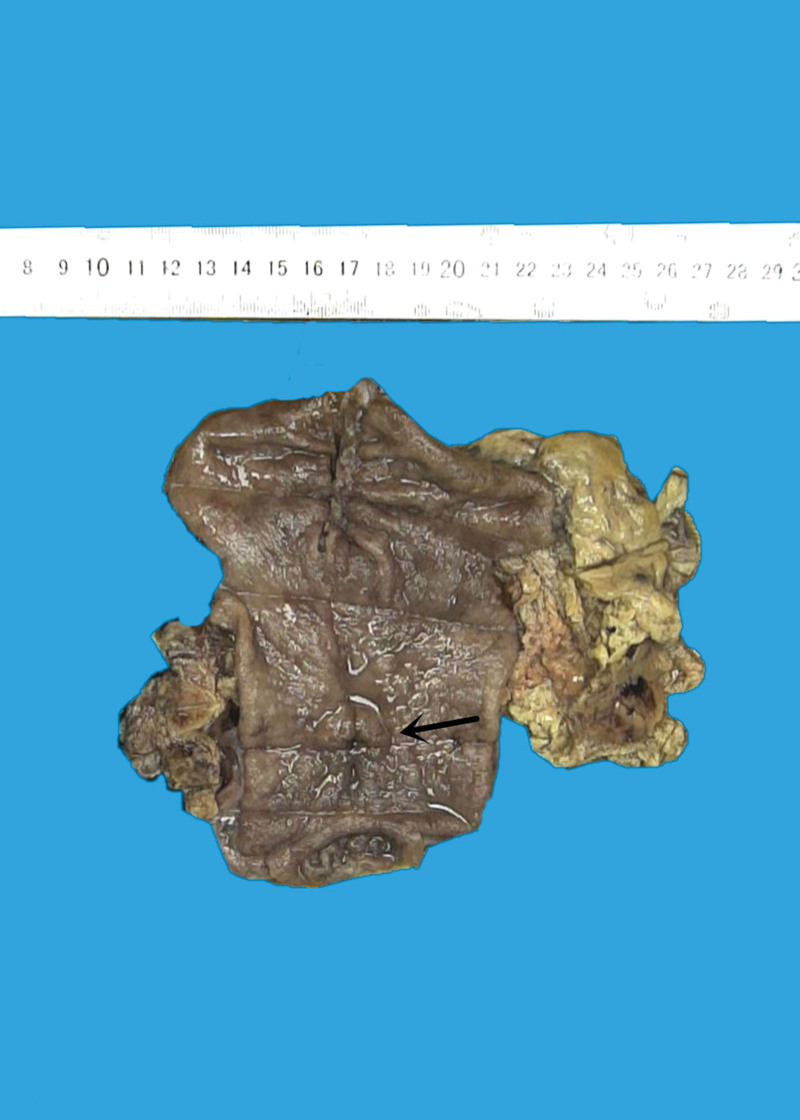
Grossly examination with naked eye, a 2.0 × 1.5 × 0.5 cm ulcerated mass was discovered in gastric antral wall.

**Figure 4.**
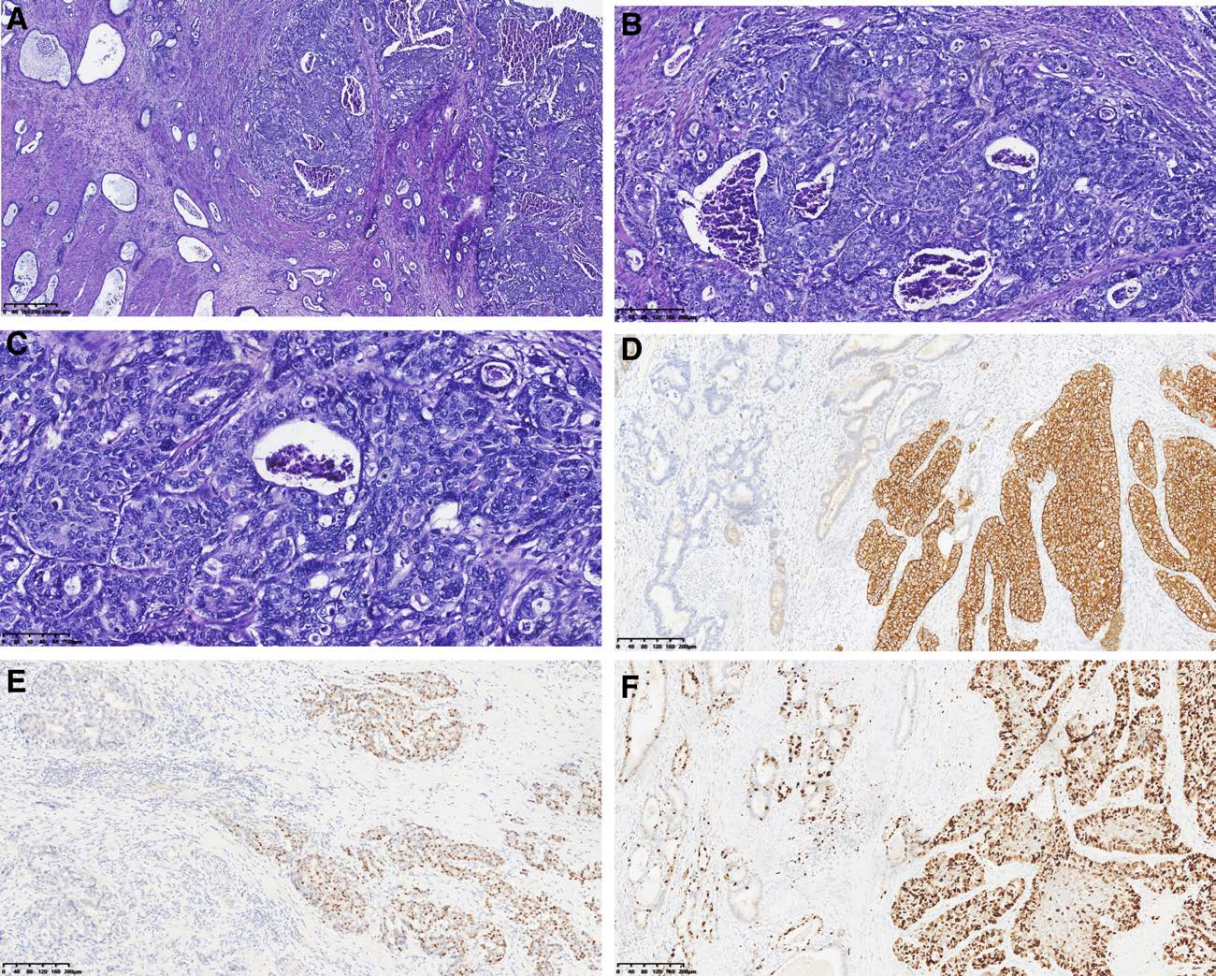
H&E staining and immunohistochemical staining of case 1. (A) Section showed neuroendocrine carcinoma (right) co-exist with adenocarcinoma within the same tumor (left). Highly atypical structures (Yellow arrows) intermingled with neuroendocrine carcinoma (Red arrows) (40× magnification; H&E). (B) LCNEC with organoid and rosette-like growth pattern, and frequent nucleoli (100× magnification; H&E). (C) Examples of large cells with a polygonal shape, ample cytoplasm, coarse chromatin, and high mitotic rate (200× magnification; H&E). (D) Immunohistochemical stain indicated that Syn was diffusely positive in the neuroendocrine carcinoma component, and it was negative in the adenocarcinoma component (100×). (E) Immunohistochemical stain indicated that INSM1 was diffusely positive in the neuroendocrine carcinoma component, and it was negative in the adenocarcinoma component (100×). (F) Ki-67 proliferative index was estimated at >80% in neuroendocrine carcinoma, and it was about 40% in adenocarcinoma (200×).

A 51-year-old male presented to our surgical department with symptoms of abdominal discomfort, acid reflux and heartburn for the past 1 month. The other past medical history was unremarkable. Esophagogastroduodenoscopy (EGD), which was performed, demonstrated an ulcerated mass of the gastric antral, which was 2.0 × 1 cm. Endoscopic biopsies of the tumor led to the initial diagnosis of adenocarcinoma. (Fig. 1). A computed tomography (CT) showed the poor filling gastric cavity. Digital GI showed the gastric sharp angle sign and the mucosal folds were irregular occurred at the lesser curvature of stomach (Fig. 2). Levels of tumor markers were shown in Table 1. After multidisciplinary team discussion, preoperative examination was considered to be malignant tumor of gastric antrum, which could be surgically removed. A laparoscopic-assisted radical gastrectomy was performed. During the operation and grossly examination with naked eye, a 2.0 × 1.5 × 0.5 cm ulcerated mass was discovered in gastric antral wall (Fig. 3). Microscopic examination showed that the large cell neuroendocrine carcinoma co-existed with adenocarcinoma within the same tumor, each of which accounting for at least 30% of the lesion. Postoperative Pathology confirmed as MANEC with stage II (pT3N0M0). The histological features of NEC were characterized by neuroendocrine appearance under light microscopy – organoid, nesting, trabecular, rosette, and palisading pattern; large cells with a polygonal shape, ample cytoplasm, coarse chromatin, and frequent nucleoli; and very high mitotic rate along with frequent of necrosis and evidence of neuroendocrine features by immunohistochemistry or electron microscopy. Immunohistochemical stains were positive for synaptophysin (Syn), chromogranin A (CgA), neural cell adhesion molecule (CD56/NCAM), INSM1, P53, and AE1/AE3 in NEC tissue; but immunohistochemical stains were negative for retinoblastoma (Rb) in mass tissue. Furthermore, the Ki-67 index was over 80% in NEC tissue (Fig. 4) and it was 40% in adenocarcinoma tissue. The neoplasm infiltrated the submucosa without spreading to the muscularis propria. No metastasis was found in 37 regional lymph nodes. The stage of the tumor was pT3N0, according to WHO2010/American Cancer Society (AJCC). The patient had an uneventful recovery following the surgery and received cisplatin-etoposide combination chemotherapy. The patient was followed-up for 1 year. The general condition was satisfactory at present, and no tumor metastasis was observed.

**Table 1:** Tumor markers of the 2 cases.

Marker	Case 1	Case 2
CEA	2.47 ng/mL	1.06 ng/mL
AFP	3.91 IU/mL	2.35 IU/mL
CA125	6.48 IU/mL	21.80 IU/mL
CA199	5.04 IU/mL	7.67 IU/mL
CA724	2.54 IU/mL	1.10 IU/mL

AFP = α-fetoprotein, CA = carbohydrate antigen, CEA = carcinoembryonic antigen.

### 2.2. Case 2

**Figure 5.**
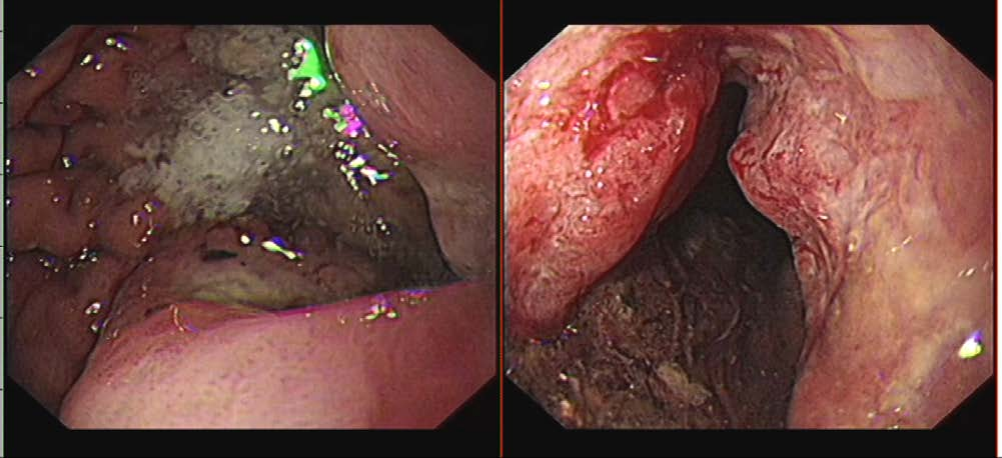
Esophagogastroduodenoscopy (EGD), which was performed, demonstrated a deep ulcerative mass occurred in stomachus cardiacus and gastric body.

**Figure 6.**
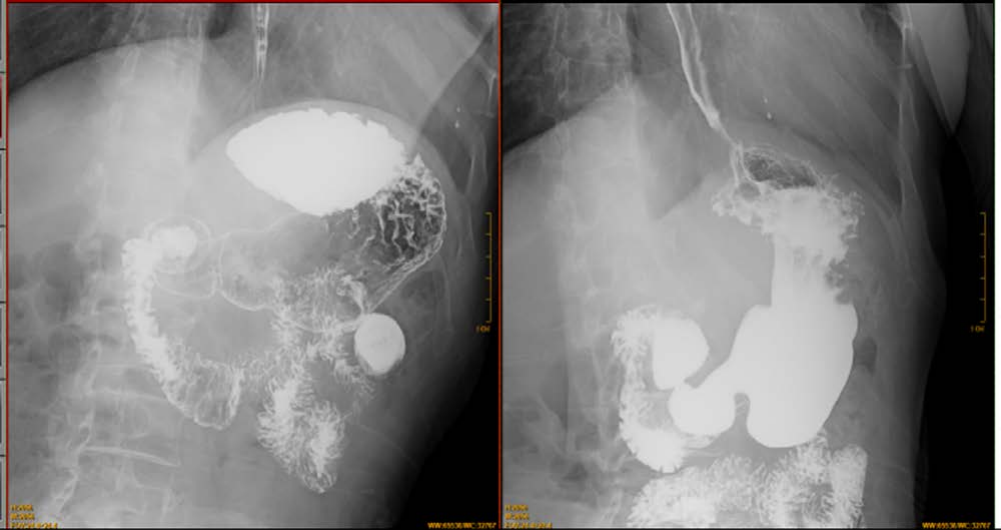
The digital gastrointestinal radiography (GI) showed a gastric carcinoma occurred in stomachus cardiacus and gastric body.

**Figure 7.**
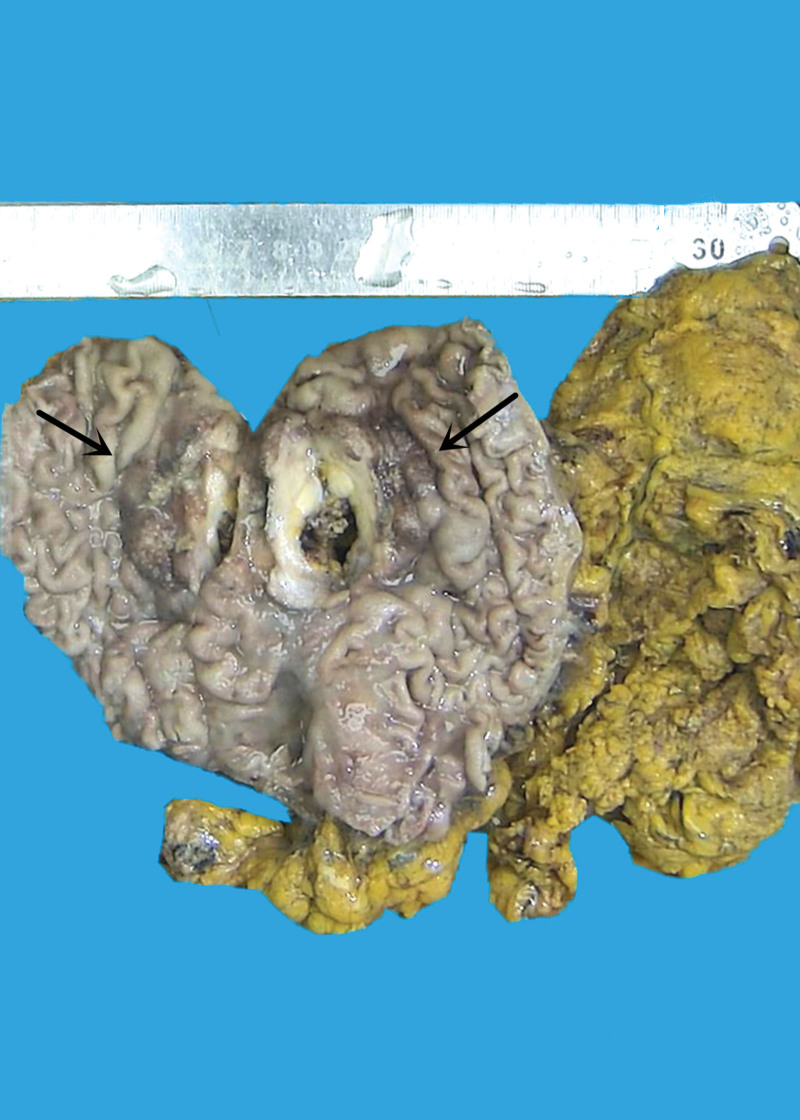
Grossly examination with naked eye, a 6.0 × 5.5 × 1.7 cm deeply ulcerative mass was discovered in stomachus cardiacus and gastric body.

**Figure 8.**
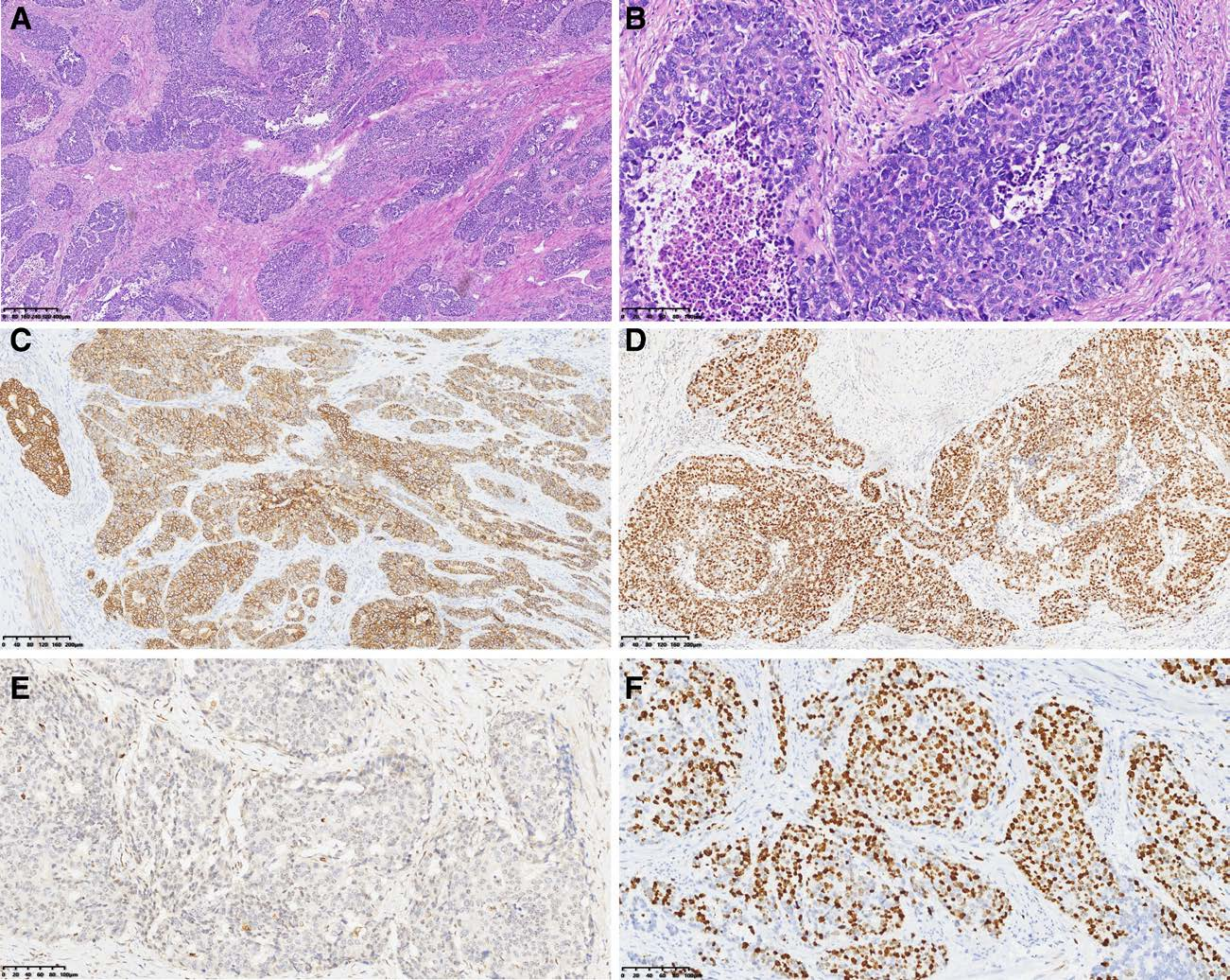
H&E staining and immunohistochemical staining of case 2. (A) Section showed a LCNEC with organoid and rosette-like growth pattern, and frequent nucleoli (40× magnification; H&E). (B) Examples of large cells with high polymorphism, large nuclei with conspicuous eosinophilic nucleoli, and high mitotic rate (100 × magnification; H&E). (C) CD56 immunohistochemical stain was diffusely positive in the neuroendocrine carcinoma (100×). (D) INSM1 immunohistochemical stain was diffusely positive in the neuroendocrine (100×). (E) Rb immunohistochemical stained was negative in adenocarcinoma carcinoma (100×). (F) Ki-67 proliferative index in neuroendocrine carcinoma was estimated at 70% (200×).

A 73-year-old male presented to our surgical department with symptoms of abdominal discomfort, acid reflux, heartburn nausea and vomiting for the past half a month. The other past medical history was unremarkable. EGD, which was performed, demonstrated a semicircular with deeply ulcerative mass in stomachus cardiacus and gastric body, and the gastroscopy diagnosis was gastric carcinoma occurred in stomachus cardiacus and gastric body (Fig. 5). Endoscopic biopsies of the tumor led to the initial diagnosis of neuroendocrine carcinoma. A CT showed a lesion in stomachus cardiacus and lesser curvature, which was consistent with malignant tumor manifestations. Digital GI showed a gastric carcinoma occurred in stomachus cardiacus and gastric body (Fig. 6). Levels of tumor markers were shown in Table 1. After multidisciplinary team discussion, preoperative examination was considered to be malignant tumor of gastric cardia and gastric body, which could be surgically removed. A laparoscopic-assisted radical gastrectomy was performed. During the operation and grossly examination with naked eye, which was performed, demonstrated a deeply ulcerative mass of 6.0 × 5.5 × 0.7 cm occurred in the gastric cardia and gastric body (Fig. 7). Postoperative Pathology confirmed it as gastric LCNEC with stage IIIA (T4aN1M0). The histological features of LCNEC were similar to the case 1. Immunohistochemical stains were positive for Syn, CgA, CD56, INSM1, P53 and AE1/AE3, but immunohistochemical stains were negative for Rb in mass tissue. In addition, the Ki-67 indexes were over 60% (Fig. 8). The neoplasm infiltrated the muscularis propria and spreading to the subserosal adipose tissue. Metastasis was found in 1 out of 26 regional lymph nodes. The stage of the tumor was pT4aN1, according to WHO2010/AJCC. The patient had an uneventful recovery following the surgery and received cisplatin-etoposide combination chemotherapy. The patient was followed up for 5 months. The general condition was satisfactory at present, and no tumor metastasis was observed.

## 3. Discussion

Neuroendocrine carcinoma is a malignant tumor derived from the diffuse neuroendocrine system (DNES). It is composed of large cell or small cell carcinoma, expressing neuroendocrine markers, which is rare in clinical practice and is <1% in all malignant tumors.^[2]^ LCNEC is a relatively rare high-grade NEC in clinical practice. It occurs frequently in the lung, gastrointestinal tract, pancreas and cervix, and often extremely aggressive with metastatic disease at the time of initial diagnosis. Even with treatment, the prognosis of these tumors is exceedingly poor. The vast majority of LCNEC were found in the lungs, followed by the gastrointestinal tract.^[3,4]^ The main clinical features of gastrointestinal LCNEC are nonspecific local symptoms involving organs, abdominal discomfort in the gastrointestinal tract, and changes in stool properties, etc. In the reported literatures, these patients with LCNEC did not show any obvious signs of manifest carcinoid syndrome, which pointed out that the LCNEC almost is always non functional neuroendocrine tumor.^[5,6]^ Clinically, gastrointestinal LCNEC usually presents as a tumor mass on CT scan, MRI and gastroscopy. In sum, there are no specific clinical symptoms and typical imaging manifestations. Endoscopically, it appears deep ulceration, semicircular with deep ulceration, or as a fungating tumor occupying the lumen. However, it is often difficult to distinguish from gastrointestinal polyps, adenoma and adenocarcinoma based on endoscopic appearance alone because of their structural similarity.^[7]^ Due to lack of specific clinical features and insensitive preoperative diagnosis, the majority of gastrointestinal LCNEC patients were considered standard gastrointestinal cancer before operation, and eventually diagnosed as gastrointestinal NEC by postoperative pathology. So it is crucial that histopathological examination and immunohistochemical staining.^[8]^

Light microscopically, the classic pathological morphological features of LCNEC include: tumor cells are distributed in nests, patches and beams, partially show a neuroendocrine pattern with rosettes, trabecules, and palisades. But the neuroendocrine morphology is not always clearly visible, only nesting of tumor cells is present in some cases. On low-power view, LCNEC looked organoid, and was similar to a carcinoid in some cases. But on higher magnification, large tumor cells (Cell diameter > 3 times resting lymphocytes) with high polymorphism, abundant eosinophilic cytoplasm, large nuclei with conspicuous eosinophilic nucleoli, and numerous mitoses (>10/2mm^2^) are obvious. Nuclei are large polymorphic (25–35 µm), with crude granular/vacuolar chromatin, enlarged and prominent nucleoli.^[9]^ Meanwhile, landscape-like necrosis was usually observed.^[10,11]^ Besides, the support of the immunohistochemical results is also essential. Some gastrointestinal LCNEC cases, contain both a neuroendocrine and an epithelial component in the same tumor, and each of which accounting for at least 30% of the lesion (MiNEN). It has been reported that the prognosis of MiNEN is better than that of the pure NEC.^[11]^ Interestingly, neuroendocrine carcinoma can co-exist with adenocarcinoma within the same tumor, forming a mixed adeno-neuroendocrine carcinoma (MANEC). There was 1 case of MANEC in this study. In 2010, the World Health Organization recognized MANEC as a very rare gastrointestinal tumor and gastric tumor,^[12]^ characterized by the presence of a neuroendocrine and an epithelial component, each of which accounting for at least 30% of the lesion.^[13]^ In most cases of gastrointestinal LCNEC combined with adenocarcinoma, LCNEC can be differentiated since adenocarcinoma is found superficially, while LCNEC is usually located in a deeper area.

The immunohistochemical profile of NEC is characteristic and helpful for the diagnosis of these neoplasms. To confirm the NEC immunological features, CgA/Syn/CD56/INSM1/Ki-67 combination is recommended. Among the 3 classic neuroendocrine markers (CgA, Syn, and CD56), there are usually 2 or 3 markers with positive expressions in LCNEC. It also supports the diagnosis of LCNEC even if there is only 1 neuroendocrine marker positive associated with the pathological characteristics of large cell carcinoma. The neuroendocrine tumors display almost 100% positive staining for Syn, which is the most sensitive marker, and about 53% positive for CgA, which is significantly lower than the positive rates of Syn and CD56. They are negative for p63, p40, CK5/6, and napsin A, which are mainly positive in squamous cell carcinomas and adenocarcinomas.^[14]^ The squamous cell carcinoma and adenocarcinoma component of MANEC was negative for the neuroendocrine markers. The Ki-67 index can indicate tumor proliferative activity, indirectly reflecting its malignant degree. Ki-67 labeling index and the mitotic count are used for classification into categories from G1 to G3.^[15]^ Poorly differentiated NEC classified as G3 NEC, either the large- or small-cell 1, which both with a high mitotic rate >20 per 10 high-power fields and Ki-67 labeling index of >20%.^[15]^

In recent years, a batch of new neuroendocrine biomarkers have emerged, such as INSM1 and somatostatin receptor subtype 2A (SSTR2A). Recently, multiple studies have shown that INSM1 has a hight expression in neuroendocrine neoplasms originated from multiple tissues, that included gastrointestinal, and is therefore a reliable marker for the diagnosis and differential diagnosis of neuroendocrine neoplasms.^[16–18]^ According to the related research reported that the most common mutated genes is TP53 in LCNEC.^[19]^ Meanwhile, inactivation of Rb gene protein is often presented in NEC.

Similar to other malignant tumors, the multidisciplinary treatment is the overall treatment strategy for LCNEC. Both surgery and chemotherapy are the main treatment methods, radiotherapy is the main treatment too; targeted drug therapy is still in the exploratory stage at present. Compared to other kinds of nonsmall cell cancer (NSCC), it needs a more active treatment method for LCNEC. Surgery is recommended for LCNEC in clinical stages i to iiiA. Surgery is the only curable and preferred treatment for LCNEC. Radical surgery makes a statistically significant improvement in survival rate. For patients with LCNEC undergoing curative surgery, even for patients in clinical stage i, it is recommended to undergo subsequent adjuvant therapy.^[20]^ The discussion of which type of chemotherapy has to be applied remained controversial for decades.^[21–23]^ In recent times a chemotherapy regimen similar to SCLC is favored.^[22,23]^ For complexed LCNEC patients, as which have non-LCNEC (NLCNEC) components, a chemotherapy regimen similar to NSCLC is preferred. Besides, another option for the treatment of LCNEC is immunotherapy. Programmed cell death 1 ligand 1 (PD-L1) expression in LCNEC was associated with poor survival, while PD-L1 expression in the tumor microenvironment seemed to have a beneficial effect.^[24]^

In a word, NEC is rare but aggressive neoplasms. LCNEC is the rarest among them. Their frequency rises more and more owing to the progress of our diagnostic methods. LCNET is very uncommon with a poor prognosis due to the aggressive nature. At the time these tumors are found, they have usually metastasized to multiple organs. This causes challenges in treatment and decreased survival rates. Earlier detection may help provide a better prognosis for future patients. More research has to be done to ameliorate our lack of knowledge about these tumors and to provide better treatment options for the patients. The existing studies revealed that radiological findings of gastrointestinal LCNEC is nonspecific, surgical resection was still the most effective therapy and that postoperative adjuvant chemotherapy could markedly improve the prognosis of patients with LCNEC. In addition, the detection of INSM1, which was a new neuroendocrine markers, played a role in the diagnosis of NEC.

## Author contributions

**Conceptualization:** Bo Wang.

**Data curation:** Yongcai Li.

**Formal analysis:** Yan Dou.

**Funding acquisition:** Yuan Si.

**Investigation:** Zhongxin Liu.

**Methodology:** Xin Xu.

**Resources:** Yuan Si.

**Validation:** Chaokang Huang.

**Writing – review & editing:** Bo Wang.
